# Maresin-like 1 Ameliorates Neuropathology of Alzheimer’s Disease in Brains of a Transgenic Mouse Model

**DOI:** 10.3390/biomedicines12122865

**Published:** 2024-12-17

**Authors:** Pallavi Shrivastava, Yan Lu, Shanchun Su, Yuichi Kobayashi, Yuhai Zhao, Nathan Lien, Abdul-Razak Masoud, Walter J. Lukiw, Song Hong

**Affiliations:** 1 Neuroscience Center of Excellence, School of Medicine, Louisiana State University Health New Orleans, 2020 Gravier St., New Orleans, LA 70112, USAyzhao4@lsuhsc.edu (Y.Z.); nathanlien01@gmail.com (N.L.); amasou@lsuhsc.edu (A.-R.M.);; 2 Department of Bioengineering, Tokyo Institute of Technology, Box B-52, Nagatsuta-cho 4259, Midori-ku, Yokohama 226-8501, Japan; 3 Organization for the Strategic Coordination of Research and Intellectual Properties, Meiji University, 1-1-1 Higashimita, Tama-ku, Kawasaki 214-8571, Japan; 4 Department of Ophthalmology, School of Medicine, Louisiana State University Health New Orleans, 2020 Gravier St., New Orleans, LA 70112, USA

**Keywords:** maresin-like, Alzheimer’s disease, neuroinflammation, neuropathogenesis, amyloid-β (Aβ), cholinergic neuron, cleaved caspase-3, M1 or M2 microglia, N1 or N2 neutrophil

## Abstract

(1) Background: Impeded resolution of inflammation contributes substantially to the pathogenesis of Alzheimer’s disease (AD); consequently, resolving inflammation is pivotal to the amelioration of AD pathology. This can potentially be achieved by the treatment with specialized pro-resolving lipid mediators (SPMs), which should resolve neuroinflammation in brains. (2) Methods: Here, we report the histological effects of long-term treatment with an SPM, maresin-like 1 (MarL1), on AD pathogenesis in a transgenic 5xFAD mouse model. (3) Results: MarL1 treatment reduced Aβ overload, curbed the loss of neurons in brains especially cholinergic neurons associated with cleaved-caspase-3-associated apoptotic degeneration, reduced microgliosis and the pro-inflammatory M1 polarization of microglia, curbed the AD-associated decline in anti-inflammatory Iba1^+^Arg-1^+^-M2 microglia, inhibited phenotypic switching to pro-inflammatory N1 neutrophils, promoted the blood–brain barrier-associated tight-junction protein claudin-5 and decreased neutrophil leakage in 5xFAD brains, and induced the switch of neutrophils toward the inflammation-resolving N2 phenotype. (4) Conclusions: Long-term administration of MarL1 mitigates AD-related neuropathogenesis in brains by curbing neuroinflammation and neurodegeneration, based on the histological results. These findings provide preclinical leads and mechanistic insights for the development of MarL1 into an effective modality to ameliorate AD pathogenesis.

## 1. Introduction

Alzheimer’s disease (AD) affects more than 6.5 million people over the age of 65 years in the United States alone [1] and 50 million people worldwide [2]. Typical neuropathological characteristics of AD include the accumulation of amyloid-β (Aβ) peptide in brain parenchyma and perivascular regions as senile plaques, and an accompanying synaptic and neuronal loss mainly in the hippocampal and cortical regions of the brain [3]. Additionally, cholinergic neuron degeneration is one of the earliest indicators of Alzheimer’s disease pathogenesis [4], and these neurons play a significant role in regulating neuroinflammation [5]. Given that the striatum is crucial for cognitive functions, contains a high density of cholinergic neurons [6], and has a markedly lower number of cholinergic neurons in human AD patients compared to non-AD control subjects [7], this report includes the study of apoptotic cholinergic neurons in the striatum in 5xFAD mice.

AD progression is greatly affected by the neuroinflammatory reactions [8]. Abnormal aggregation of Aβ is identified as one of the primary factors [9]. Recognition of the significance of neuroinflammation in AD has brought more attention to specialized pro-resolving mediators (SPMs). These types of mediators effectively promote the resolution of inflammation [10]. One class of these compounds are maresins, which were discovered by Serhan et al. [11,12]. Maresins and the related maresin-like (MarL) mediators [13] are derived from essential ω-3 docosahexaenoic acid (DHA) by the action of endogenous enzymatic systems [12- or 15-lipoxgenase (LO) for maresins; and 12/15-LO combined with a cytochrome P450 CYP4F3 for MarL]. Maresin-1 (7*R*,14*S*-dihydroxy-4*Z*,8*E*,10*E*,12*Z*,16*Z*,19*Z*-DHA) reduces neuroinflammation, mitochondrial damage, and neuronal death, while enhancing neural functional recovery, phagocytosis of Aβ, and the switch in macrophage phenotype to the anti-inflammatory M2 type [14,15]. Maresin-1 improves the pathological condition of experimental autoimmune encephalomyelitis [16]. In Aβ42-treated C57BL/6 mice, cognitive decline and neuroinflammation is alleviated by maresin-1 treatment [17]. Similarly, in another AD mouse model (App^NL-G-F/NL-G-F^), microglial activation has been significantly reduced by the intranasal instillation of a group of SPMs, including maresin-1 [18].

In contrast, the maresin-like compound MarL1 (14*S*,22-dihydroxy-docosa-4*Z*,7*Z*,10*Z*,12*E*,16*Z*,19*Z*-hexaenoic acid) promotes the release of the regenerative angiogenic growth factor HGF and reduces the expression of inflammatory cytokine TNFα in macrophages in vitro [13]. MarL1 recovers macrophage functions in the elevation of migration of epithelial cells and fibroblasts in scratch-wounded monolayer cultures, as well as stem cell transmigration [13]. Despite the growing evidence for pro-resolving and neuroprotective effects of maresin-1 and MarLs, the underlying mechanisms require further investigation for their therapeutic development. In the present study, we hypothesize that long-term administration of MarL1 administration could mitigate AD-related brain neuropathogenesis by curbing neuroinflammation and neurodegeneration.

The 5x familial Alzheimer’s disease (5xFAD) transgenic mouse overexpresses humanized sequences of five AD-linked mutations, including the Swedish (K670N/M671L), Florida (I716V), and London (V717I) mutations in APP and the M146L and L286V mutations in PSEN1 under the regulation of the neuron-specific thy1 promoter [19]. The most prominent feature of 5xFAD mice is that they present AD amyloid pathology and neuron loss at young age [19,20].

Neuroinflammation starts as early as 2–3 months of age in 5xFAD mice [19]. Pro-inflammatory M1-phenotype microglia are major contributors and indicate the occurrence of the neuroinflammation [21,22,23]. As resident immune cells of the central nervous system, resting microglia continuously survey the microenvironment of the brain [24]. Under adversary conditions, microglia become activated and transfer to either the M1 or M2 phenotype [25]. M1-type microglia adopt an amoeboid shape [26], are highly phagocytic, and express CD68 at a high level [27]. In contrast, alternative M2 microglia play an important role in immuno-resolution and repair processes during an injury, exhibiting neuroprotective effects [28,29]. The switching between M1 and M2 phenotypes relies on the severity and progression of the disease [30,31].

Neutrophils are generally scarce in healthy brains due to the brain–blood barrier (BBB) [32]. Under neuroinflammatory conditions, neutrophils are observed to promote the BBB damage and transmigrate into brain parenchyma [33,34,35]. There have been reports on increases in the neutrophil population in different models of AD [36,37]. Neutrophils penetrate the brain parenchyma by breaching the BBB and move toward amyloid plaques in the 5xFAD mouse model [38]. Analogous to the conventional categorization of macrophages or microglia into two major types, M1 and M2 phenotypes, neutrophils have been categorized into pro-inflammatory N1 [39] and anti-inflammatory N2 subpopulations [40]. Their transcriptomic profiles are distinctive [39]. N1 and N2 neutrophils function differently in neuroinflammation [41]. N1 neutrophils exhibit a remarkably increased production of reactive oxygen species (ROS) as well as nitric oxide (NO). By contrast, N2 neutrophils demonstrate increased expression of Arginase 1 (Arg1) [39,41]. We histologically determine the possible occurrence of N1 and N2 neutrophils in brains of 5xFAD mice with and without treatment as well as of wildtype control mice in this study.

## 2. Materials and Methods

### 2.1. Animals

The Louisiana State University Health Science Center (LSUHSC) IACUC committee approved all the animal procedures, which are consistent with American Veterinary Medical Association guidelines. Mice were maintained at LSUHSC at a controlled temperature of 25 ± 2 °C and 50–65% humidity with a fixed 12:12 h light/dark cycle. We used a widely used model of AD—5xFAD transgenic mice (Jackson Laboratory, Bar Harbor, ME, USA) with a C57BL/6J genetic background (MMRRC Strain #034840-JAX)—and compared it with wildtype (WT) control mice (C57BL/6J) [42].

### 2.2. Intranasal Treatment with Maresin-like 1

Male mice at the age of 1.5 months were randomly selected and equally divided into the following three groups: (1) WT (C57BL/6J mice), (2) 5xFAD mice treated with vehicle, and (3) 5xFAD mice treated with MarL1 plus vehicle. The MarL1 or vehicle was administered by the intranasal route 3 times per week to each mouse from 1.5 months to 9 months of age. Briefly, each mouse was temporarily immobilized for about 60 s by a slight anesthetization using isoflurane inhalation. While the mouse was immobilized, it was held in the supine position and its nostrils were then instilled with 3 µL/nostril of MarL1 (100 ng per mouse for each administration) dissolved in the vehicle (0.05% dimethyl sulfoxide [DMSO] in sterile 0.9% saline) or with 3 µL of the vehicle alone. The mouse was held at the same position for 20 more seconds to ensure liquid intranasal intake [18,43,44,45]. The mice were sacrificed at the age of 12.5 months.

### 2.3. Harvesting of Murine Brains for Immunohistology

The mice were anesthetized with 5% isoflurane and subsequently perfused transcardially with 4% paraformaldehyde (PFA). A change in liver color from deep red to a lighter shade was used to indicate adequate perfusion. The mice were then decapitated, and the brains were quickly removed and immersed in 4% PFA overnight at 4 °C. After 24 h, the brains were placed in a 15% sucrose solution for 12 h, followed by a 30% sucrose solution until they sank. The brain tissue was then embedded in optimal cutting temperature compound (OCT) and cryo-molds for cryo-sectioning. The coronal section of the brain was serially cut from rostral to caudal at 20 μm thickness on an HM550 cryostat (Microm-HM 550, Thermo Fisher Scientific, Kalamazoo, MI, USA). These sections were then mounted on Superfrost Plus glass slides (VWR, Radnor, PA, USA), and sections from the cortex and hippocampus regions were selected for immunofluorescence.

### 2.4. Immunofluorescence Staining

The mounted brain sections (containing cortex and hippocampus) were washed twice in phosphate-buffered saline (PBS), followed by two washes in PBS-T (PBS containing 0.05% Triton-X). The sections were then incubated in blocking buffer (1% BSA + 0.5% Trition-X + 0.02% Tween-20 in 1X PBS) at room temperature for 60 min. The sections were then treated with the following antibodies: NeuN (1:500, rabbit, Cat #: 12943S, Cell Signaling Technology, Danvers, MA, USA), amyloid-β (MOAB-2, 1:500, mouse host, monoclonal 6C3, Cat #: MABN254, Millipore Sigma, Temecula CA, USA), Gr-1 (Ly-6G/Ly-6C monoclonal antibody, 1:200, rat host, Invitrogen, Memphis, TN, USA), ionized calcium-binding adapter molecule-1 (Iba-1, 1:500, rabbit, Cat #: 019-19741, Fujifilm Wako, Temecula, CA, USA), iNOs (1:200, rat, Invitrogen eBioscience, Carlsbad, CA, USA), Arg-1 (1:500, goat, Cat #: ab60176, Abcam, Boston, MA, USA), anti-choline acetyltransferase (1:500, ChAT, goat, Cat #: AB144P, Millipore Sigma, Temecula, CA, USA), cleaved caspase-3 (cleaved-caspase-3, 1:500, rabbit, Cat #: 9661, Cell Signaling Technology, Danvers, MA, USA) antibody, CD68 (1:500, mouse, Cat #: sc-70761, Santa Cruz Biotechnology, Dallas, TX, USA), and claudin-5 (1:500, mouse, Cat #: sc-374221, Santa Cruz Biotechnology, Dallas, TX, USA). The sections were then incubated with appropriate secondary antibodies (Alexa Fluor 488, 568, or 594; Invitrogen) compatible with the aforementioned primary antibodies, followed by the incubation with DAPI (1:10,000 dilution). A total of three to four sections of brain per slide from each of five to eight mice per group were used for the histological study. Co-stained (yellow-colored) cells by two protein/peptide biomarkers (green-colored and red-colored) were quantified by cell counting per field in 20× zstack image or Pearson’s coefficients in 10× image per field obtained from ImageJ analysis (Version: 1.54J, National Institutes of Health, Bethesda, MD, USA).

### 2.5. Image Quantification

After immunofluorescence staining, fluorescent images were captured on ECHO Revolve fluorescent microscope using 4×, 10×, 20×, and 40× objectives, and mean intensity of fluorescence as well as number of immunoreactive cells were quantified using ImageJ software version: 1.50d. The results were expressed in mean fluorescence intensity (MFI) per field in a 10× image, as we conducted previously [46]. The counting of co-localized immune-positive cells for two markers was performed manually using the cell counter application of ImageJ. The researchers performed this study blindly. The microglial morphology was assessed by Sholl analysis based on circularity by ImageJ. The z-stack images were condensed into a maximum intensity projection image over which concentric circles were drawn (concentric circles plugin, Image J), centered on the soma, beginning at 5.5 μm radii and increasing 2 μm with every circle. Sholl analysis was manually performed for each cell by counting the number of intersections between microglia branches and each increasing circle to create a Sholl plot and based on circularity and processes the microglia were divided into groups. The microglia were divided into 4 groups based on the number of branches emerging from soma: 1. Ramified microglia; 2. Partially ramified; 3. Partially amoeboid; and 4. Fully amoeboid microglia. We counted the branches of microglia attached to cell body (soma) by drawing a circle around soma and counted the numbers of intersecting points between concentric circles to evaluate the complexity of microglia. The cell counts of microglia were obtained in each group.

### 2.6. Thioflavin S Staining and Analysis of Plaques

Thioflavin S (Cat #: T1892, Sigma-Aldrich, Louis, MO, USA) serves as a fluorescent dye for the detection of Aβ plaques in brain tissue [47]. The thioflavin S-stained Aβ plaques appear bright green under fluorescence microscopy. Briefly, brain sections (20 μm thickness) were incubated in 1% thioflavin S solution in deionized water for 10 min, washed with running water carefully for 5 min, then incubated in 1% acetic acid for 15 min and rinsed again. The slides were dried by placing them on paper towels for few minutes and then dehydrated in 70%, 80%, 95%, and 100% ethanol sequentially; transferred to xylene; and then mounted with Permount mounting medium and dried overnight in the dark. Thioflavin S-positive plaques were determined from the images taken by Discover-ECHO Revolve fluorescence microscope in a single plain at 4× magnification. The images were subjected to threshold processing (Otsu) using ImageJ, and the total number of plaques based on size (less than and greater than 100 μm) were analyzed in the cortical and hippocampal regions. For each animal, 6 fields from the cortex and from the hippocampus were imaged and analyzed.

### 2.7. Statistical Analysis

Results are reported as mean ± standard error of the mean (SEM). GraphPad Prism 9.0 (GraphPad, Boston, MA, USA) was employed for graph creation and statistical analyses. For comparisons among more than two groups with a single factor, one-way ANOVA followed by Tukey’s test was used. For comparisons among more than two groups with two factors, two-way ANOVA with Geisser–Greenhouse correction and mixed-effects model followed by Tukey’s multiple comparisons test were performed. For the comparison between two groups, an unpaired Student’s *t*-test was used. Normal data distribution was assessed using the Kolmogorov–Smirnov or Shapiro–Wilk test (α = 0.05). If the normality test failed, data were analyzed using the Kruskal–Wallis test with Dunn’s post-hoc test.

## 3. Results

### 3.1. Maresin-like 1 Reduces Aβ Overload and Curbs Neuronal Population Loss in Brain Hippocampi of 5xFAD Mice

**Figure 1:**
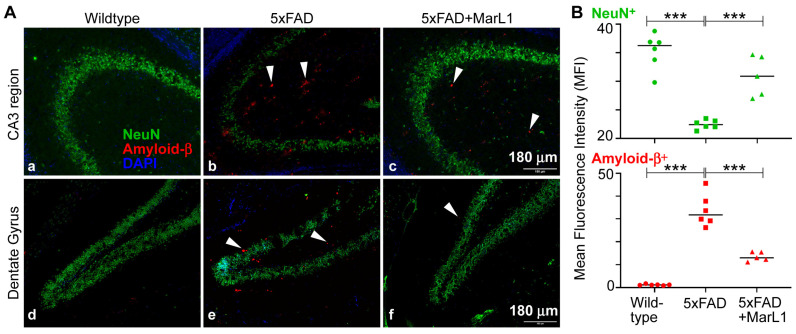
MarL1 treatment ameliorated AD neuropathology in brains of 5xFAD mice. (**A**) Immunostaining of NeuN (green) and Amyloid-β_1–42_ (red) in CA3 and dentate gyrus (DG) of hippocampus. White arrows mark some Aβ_1–42_ deposition in hippocampal regions. Panels a–f: 10× magnification; scale bar: 180 μm. (**B**) Quantification of NeuN^+^ and Amyloid-β_1–42_^+^ staining intensities of hippocampus (mean fluorescence intensity—MFI). Data are means ± SEM. Wildtype *n* = 6, 5xFAD *n* = 6, and 5xFAD+MarL1 *n* = 5. *** *p* < 0.001.

Amyloid beta deposition is most prominent in the hippocampus and cortex in the brain, especially in 5xFAD mice, as the Aβ deposition starts as early as 2 months of age [19,48]. Here, we examined the effect of long-term intranasal instillation of MarL1 on Aβ deposition and neuronal population in the hippocampus of 5xFAD mouse brains. We found a significantly more Aβ plaques in the hippocampus of the 5xAD mice than in the same brain regions in the wildtype mice (Figure 1, *p* < 0.001), and significantly fewer Aβ_1–42_ plaques in the MarL1-treated 5xFAD mice than in the untreated 5xFAD mice (Figure 1, *p* < 0.001). We also evaluated the cerebral plaque in hippocampal and cortical sections by staining them with thioflavin S (Thio-S) dye that detects the β-pleated sheet of the amyloid plaques [47]. The level of plaque deposition was quantified in the hippocampus and cortex by determining the number of plaques of size > 100 μm^2^ and size < 100 μm^2^ [49,50,51]. We found a markedly greater number of plaques in the cortex and hippocampus of 5xFAD mice than in the wildtype mice (Figure S1). The numbers of plaques of each size category were markedly lower in both cortex and hippocampus of the MarL1-treated 5xFAD mice than in the same brain regions in the vehicle treated 5xFAD mice (Figure S1, *p* < 0.001). We found that Iba-1^+^ microglia engulfed the Aβ plaques and that those microglial cells formed clusters near Aβ plaques in 5xFAD mice based on the histological examination (Figure S2).

We also examined Aβ-related neuronal losses by co-staining brain sections with MOAB-2 and with NeuN antibody. The NeuN^+^ neuronal population was significantly lower in the 5xFAD mice than in the wildtype mice (Figure 1B, *p* < 0.001). MarL1 treatment rescued the NeuN^+^ neuronal population in hippocampus (Figure 1B, *p* < 0.001), suggesting that MarL1 had a neuroprotective effect against AD pathogenesis in the brains of 5xFAD mice at histological level.

### 3.2. Maresin-like 1 Treatment of 5xFAD Mice Improves the Survival of Cholinergic Neurons and Decreased Cleaved-Caspase-3-Mediated Apoptotic Degeneration

Cholinergic neurons play the crucial roles on maintaining normal cognitive function through the neurotransmitter acetylcholine (Ach) [52]. These neurons broadly distribute throughout the brain. Striatum is one of the main areas where cholinergic neurons are found [53]. Striatum has the greatest levels of ACh in the brain [6,54]. The loss, degeneration, or dysfunction of cholinergic neurons in the brain leads to cognitive deterioration, a characteristic feature of AD [55,56].

**Figure 2:**
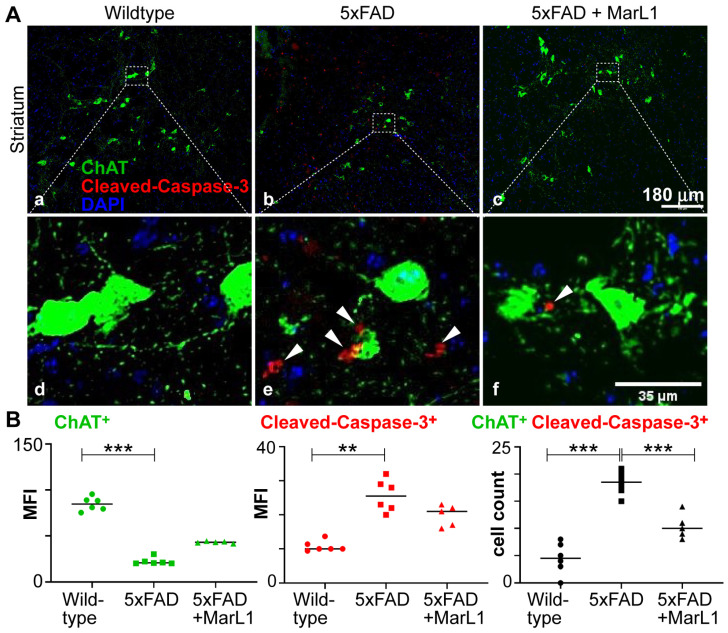
MarL1 protected cholinergic neurons (ChAT^+^) and inhibited apoptotic cleaved caspase-3 activity in brains of 5xFAD mice. (**A**) Immunostaining of ChAT (green) and cleaved caspase-3 (red) in striatum (Panels a–c): 10× magnification; scale bar: 180 µm. White arrows mark cleaved caspase-3^+^ cholinergic neurons in zoomed-in images (Panels d–f). Scale bar: 35 µm. (**B**) Quantification of ChAT and caspase-3 in striatum. Left: mean fluorescence intensity MFI for ChAT^+^; middle: MFI for cleaved caspase-3^+^; right: count of cells stained positive for both ChAT and cleaved-caspase-3. Data are means ± SEM. Wildtype *n* = 6, 5xFAD *n* = 6, and 5xFAD+MarL1 *n* = 5. *** *p* < 0.001 and ** *p* < 0.01.

Here, we investigated whether MarL1 treatment could curb the AD-associated decline in the cholinergic neuronal population in 5xFAD mice. We immunostained striatal brain sections with an antibody specific to choline acetyltransferase (ChAT), an enzyme that catalyzes the synthesis of acetylcholine at cholinergic synapses, thus selectively marks cholinergic neurons [57,58]. We conducted the ChAT immunostaining together with cleaved caspase-3 antibody to quantify the apoptotic ChAT^+^ neuronal population in the brain (Figure 2A). We found that the ChAT^+^ neuronal population was significantly smaller in the 5xFAD mice than in their wildtype littermates (*p* < 0.001, one-way ANOVA followed by Tukey’s post-hoc test), whereas MarL1 treatment inhibited this decline in the ChAT^+^ population in 5xFAD mice compared to vehicle-treated 5xFAD mice (*p* < 0.001, one-way ANOVA followed by Tukey’s post-hoc test) (Figure 2B) in the striatum region. The number of apoptotic cleaved caspase-3^+^ cells was also lower in the striatum region of 5xFAD mice treated with MarL1 than with vehicle (Figure 2B, *p* < 0.001, one-way ANOVA followed by Tukey’s post-hoc test). Notably, when assessing the normal distribution for ChAT and cleaved caspase-3 staining individually (Figure 2B, left and middle panels), wildtype and 5xFAD+MarL1 passed the normality test of ChAT with both Kolmogorov–Smirnov and Shapiro–Wilk methods at α < 0.05, whereas 5xFAD did not pass. Moreover, 5xFAD and 5xFAD+MarL1 passed the normality test of cleaved caspase-3 with both Kolmogorov–Smirnov and Shapiro–Wilk methods at α < 0.05, whereas wildtype did not pass. Although one-way ANOVA can be used for non-normal distributions under specific conditions, to ensure definitive results, we reanalyzed ChAT and cleaved caspase-3 staining individually using the Kruskal–Wallis test followed by Dunn’s test. This non-parametric analysis (*p* < 0.001 for ChAT in wildtype vs. 5xFAD, *p =* 0.0014 for cleaved caspase-3 in wildtype vs. 5xFAD) validated the significant differences between wildtype and 5xFAD identified in one-way ANOVA but showed no statistically significant difference between 5xFAD and 5xFAD+MarL1. Therefore, MarL1 treatment might not have a statistically significant effect on ChAT or cleaved caspase-3 staining individually. However, an experiment with a larger number of animals should be conducted in the future to obtain more statistically definitive results. Additionally, the number of ChAT^+^ cleaved caspase-3^+^ co-stained cells in the striatum was significantly greater in 5xFAD mice compared to wildtype controls (*p* < 0.001). In contrast, these counts were significantly reduced in 5xFAD+MarL1 mice compared to 5xFAD mice (*p* < 0.001), with the data meeting the normality criteria. Thus, these data were only analyzed by one-way ANOVA followed by Tukey’s post-hoc test. The ChAT^+^ cleaved caspase-3^+^ co-stain results suggest that MarL1 treatment may be effective in protecting cholinergic neurons from apoptosis and restoring their population in the brains of 5xFAD mice.

### 3.3. Maresin-like 1 Attenuates the Pro-Inflammatory M1 Phenotypic Switching of Microglia by Inhibiting Iba-1^+^CD68^+^ Microglia in Brains of 5xFAD Mice

**Figure 3:**
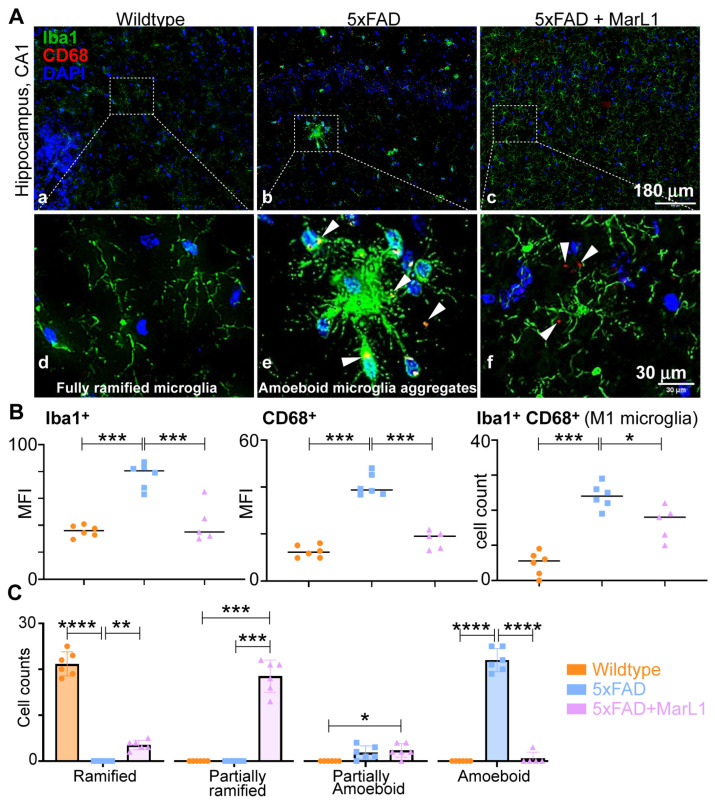
MarL1 suppressed pro-inflammatory M1 phenotype polarization of microglia in brains of 5xFAD mice. (**A**) Immunostaining of microglia with Iba-1 (green) and CD68 (red) in CA1 region of hippocampus from 5xFAD transgenic mice (Panels a–c: 10× magnification; scale bar: 180 µm. Panels d–f: zoomed-in images; scale bar: 30 µm). White arrows mark Iba-1^+^CD68^+^ microglia. (**B**) Quantification of Iba-1^+^ and CD68^+^ in hippocampus. Left: mean fluorescence intensity MFI of Iba-1^+^; middle: MFI of CD68^+^; right: count of microglia stained positive for both Iba-1^+^ and CD68^+^. (**C**) Quantification of microglia based on phenotype characterization (ramified, partially ramified, partially amoeboid, amoeboid) in hippocampus. Data are means ± SEM. Wildtype *n* = 6, 5xFAD *n* = 6, and 5xFAD+MarL1 *n* = 5 for (**B**). Wildtype *n* = 6, 5xFAD *n* = 6, and 5xFAD+MarL1 *n* = 6 for (**C**). **** *p* < 0.0001, *** *p* < 0.001, ** *p* < 0.01, and * *p* < 0.05.

We investigated the effect of MarL1 on M1 phenotypic switching of microglia in brains of 5xFAD mice by immunohistological assessment of the microglial phenotype using the markers Iba-1 and CD68 for M1 microglia [27,59,60,61] in the brain (Figure 3A). Significantly greater immunoreactivities of Iba-1 were found in 5xFAD mice than in wildtype mice in hippocampus region (Figure 3B, *p* < 0.001), while the immunoreactivities of Iba-1 were significantly lower in MarL1-treated mice than in vehicle-treated 5xFAD mice (Figure 3B, *p* < 0.001).

We also quantified the levels of CD68 (scavenger receptor Class D), a phagocytic microglial marker on Iba-1^+^ microglial cells, in co-stained brain coronal sections [62]. The activation of CD68^+^ microglial cells was 3.25-fold higher in 5xFAD mice than in their wildtype littermates (Figure 3B, middle panel, *p* < 0.001), but this activation was significantly attenuated by 57% in the MarL1-treated 5xFAD mice (Figure 3B, middle panel, *p* < 0.001). The Iba-1^+^CD68^+^ cell count was significantly higher in 5xFAD brains than in wildtype controls (Figure 3B, right panel, *p* < 0.001), and was significantly lower in MarL1-treated 5xFAD mice than in vehicle-treated 5xFAD mice (Figure 3B, right panel, *p* < 0.05).

Microglia can be phenotypically categorized based on their circularity (i.e., the length of their processes) into different stages of activation ranging from ramified microglia (microglia at the resting stage, S0) to amoeboid microglia (microglia at the activated-stage, S1). Inter-stages also exist between S0 and S1 in the form of partially ramified microglia (having fewer processes) and partially amoeboid microglia (having 1–2 processes with a round shape) [63,64,65]. Notably, we observed an unusual phenomenon in the microglial phenotype in 5xFAD mice. We found that the microglial cells aggregated in the hippocampus and cortex to form a “cluster of cells” similar to those found near Aβ plaques and those microglia were partially or fully amoeboid in shape in the 5xFAD mice but did not show this morphology in the wildtype mice. We also observed some microglial aggregates in the cortex, but not in the hippocampus, of the MarL1-treated 5xFAD mice. The MarL1 treatment group showed more partially ramified microglial phenotypes than the vehicle-treated 5xFAD mice (Figure 3A).

We also quantified the numbers of microglial cells based on the morphology observed in Iba-1-immunostained sections. The number of ramified microglia was greater in the wildtype mice than in the 5xFAD and MarL1-treated mice (Figure 3C). The number of partially ramified microglia was significantly higher in the MarL1-treated 5xFAD mice than in the vehicle-treated 5xFAD or wildtype mice (Figure 3C, *p* < 0.001). The amount of amoeboid microglia was significantly higher in the vehicle-treated 5xFAD mice than in the wildtype (Figure 3C, *p* < 0.001) and MarL1-treated 5xFAD mice (Figure 3C, *p* < 0.001). These histological results suggest that the transition of microglia from the ramified (S0) morphology to activated amoeboid (S1) morphology was greater in the 5xFAD mice than in the wildtype mice, while treatment with MarL1 attenuated that morphological transition.

### 3.4. Maresin-like 1 Curbs AD Pathogenesis-Associated Decline in M2 Microglial Population with an Anti-Inflammatory Alternatively Activated Phenotype in Brains of 5xFAD Mice

**Figure 4:**
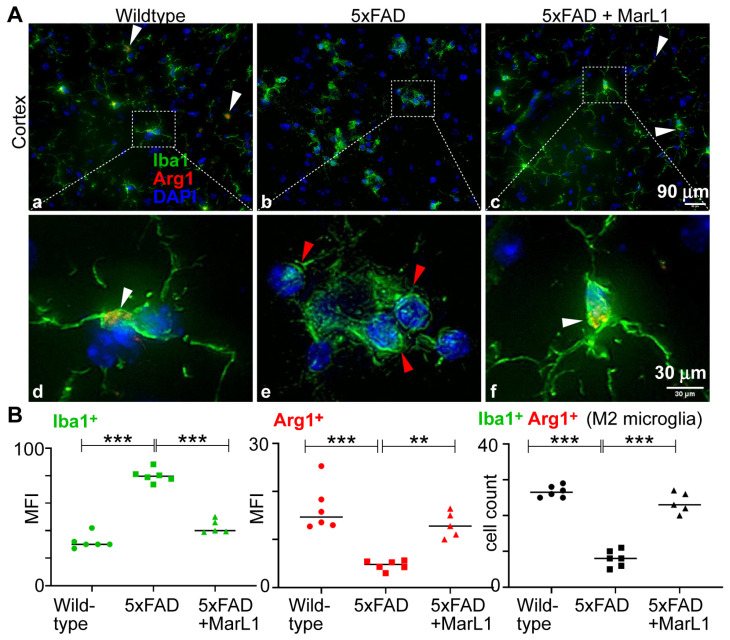
MarL1 promoted anti-inflammatory M2 phenotype polarization of microglia in brains of 5xFAD mice. (**A**) Immunostaining of microglia with Iba-1 (green) and Arg1 (red) in cortex (Panels a–c: 20× magnification; scale bar: 90 µm. Panels d–f: zoomed-in images; scale bar: 30 µm). White arrows mark Iba1^+^Arg1^+^ microglia. Red arrows mark microglial aggregation in cortex of 5xFAD mice. (**B**) Quantification of Iba-1 and Arg1 in cortex. Left: mean fluorescence intensity MFI of Iba1^+^; middle: MFI of Arg1^+^; right: count of microglia stained positive for both Iba1 and Arg1 in cortex. Data are means ± SEM. Wildtype *n* = 6, 5xFAD *n* = 6, and 5xFAD+MarL1 *n* = 5. *** *p* < 0.001 and ** *p* < 0.01.

We determined the effect of MarL1 on the microglial phenotypic switch between M1 and M2 by investigating the M2 population in cortical brain sections. Immunostaining brain sections with Iba-1 and Arg-1, a biomarker for M2 microglia [66] (Figure 4A), revealed a significantly higher mean fluorescence intensity for Arg-1 in the brains of wildtype mice than of 5xFAD mice (Figure 4B, *p* < 0.001), but the population was significantly greater in MarL1-treated 5xFAD mice than in vehicle-treated 5xFAD mice; that is, the MarL1 treatment significantly restored the Arg1^+^Iba-1^+^ microglial population in 5xFAD mice (Figure 4B, *p* < 0.001). These histological results suggest that MarL1 treatment significantly shifted the polarization of the microglial population from M1 to M2, suggesting that MarL1 has a capacity for resolution of inflammation that could contribute to curbing AD pathogenesis.

### 3.5. Maresin-like 1 Treatment Promotes the Expression of BBB-Associated Tight-Junction Protein Claudin-5, Decreases Infiltration of Neutrophils in 5xFAD Brains, and Induces the Switch of Neutrophils Toward the Inflammation-Resolving N2 Phenotype

Conventionally, AD has been viewed mainly as a neurodegenerative disorder. However, new reports suggest that dysfunction of the blood–brain barrier link to neuroinflammation of the brain, which in close association with peripheral inflammatory signals, also plays a significant role in AD [33,34,35,67,68]. Patients with early cognitive decline or mild cognitive impairment have been reported to show BBB breakdown in the hippocampus (CA1, CA3, and dentate gyrus) and parahippocampal gyrus, areas crucial for memory and learning [69]. As a result, the BBB can no longer effectively clear Aβ, and that Aβ then accumulates in the brain and blood vessels [70,71], leading to increased expression of adhesion molecules on brain blood vessels and the release of inflammatory substances that potentially promote leukocyte recruitment [70].

**Figure 5:**
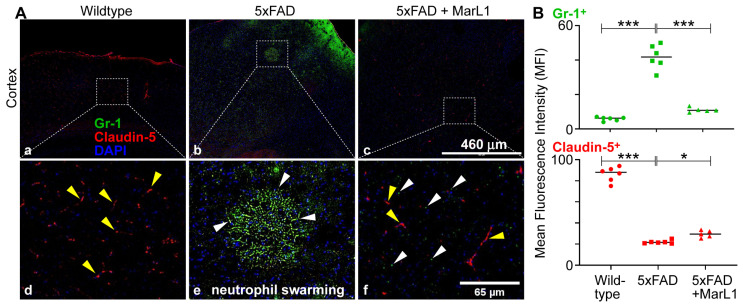
MarL1 attenuated the AD-associated compromise of blood–brain barrier tight-junctions as well as neutrophil infiltration into brains of 5xFAD mice. (**A**) Immunostaining of Gr-1 (green) for neutrophils and claudin-5 (red) for tight-junctions of the vasculatures in cortex. Panels a–c show images from cortex (4× magnification; scale bar: 460 µm). Panels d–f show zoomed-in images; scale bar: 65 µm. White arrows mark some Gr-1^+^ cells outside the vasculature in parenchyma in zoomed-in images. Yellow arrows mark some claudin-5^+^ vasculatures. Neutrophil swarming is evident in Panels b and e. (**B**) Quantification of Gr-1^+^ and claudin-5^+^ in MFI in cortex. Data are means ± SEM. Wildtype *n* = 6, 5xFAD *n* = 6, and 5xFAD+MarL1 *n* = 5. *** *p* < 0.001 and * *p* < 0.05.

When activated, resident immune cells in brains, such as microglia, can release tissue danger signals, cytokines, and chemokines that recruit leukocytes from the peripheral circulation to inflamed areas in brains. Both experimental and clinical evidence shows that neutrophils and T-cells migrate into the brain in AD [72,73,74,75]. A study using two-photon laser scanning microscopy revealed that vascular deposition of Aβ in brains of 5xFAD mice facilitates intraluminal adherence and movement of neutrophils in brain blood vessels [38,76]. Neutrophils were discovered to selectively leak into the parenchyma of the regions with Aβ deposits. [38,76]. We explored the possibility of neutrophil infiltration in the brain by immunostaining cortical sections with Gr-1 (a marker for myeloid differentiation, present primarily in neutrophils and transiently in monocytes/macrophages) and claudin-5 (a tight-junction protein present in endothelial cells of vessels in brain for vasculature), as shown in Figure 5A. We found a significantly greater influx of neutrophils in 5xFAD mice than in wildtype mice (Figure 5B, *p* < 0.001). The MarL1 treatment significantly reduced this influx compared to vehicle-treated 5xFAD mice (Figure 5B, *p* < 0.001). We also observed a phenomenon called “neutrophil swarming” in 5xFAD mice, which is the accumulation of large numbers of neutrophils to neutralize large microbes and clusters of microbes in a targeted region [77,78]. Here, we noticed a massive number of neutrophil aggregations in the cortex of 5xFAD mice brains. One possibility is that neutrophil swarming may occur due to the presence of Aβ plaques in the cortex of 5xFAD mice. Here, we report the first evidence for “neutrophil swarming” in the cortex of the brains of 5xFAD mice. We did not observe neutrophil swarming in MarL1-treated 5xFAD mice, although the neutrophil clusters that are not swarming were evident in the cortical sections (Figure 5A).

BBB integrity was further assessed using the tight-junction protein marker claudin-5, which is critical for preserving the integrity of the endothelial cells of brain blood vessels, and staining endothelial cells in the blood vessels in the brain [79]. The mean claudin-5 intensities were significantly lower in 5xFAD mice than in wildtype mice. The MarL1 treatment restored the BBB integrity by increasing the claudin-5 levels in brain blood vessels in 5xFAD mice (Figure 5B). These results suggest that MarL1 treatment may provide a protective effect on the BBB integrity and decrease the infiltration of immune cells into the brain.

**Figure 6:**
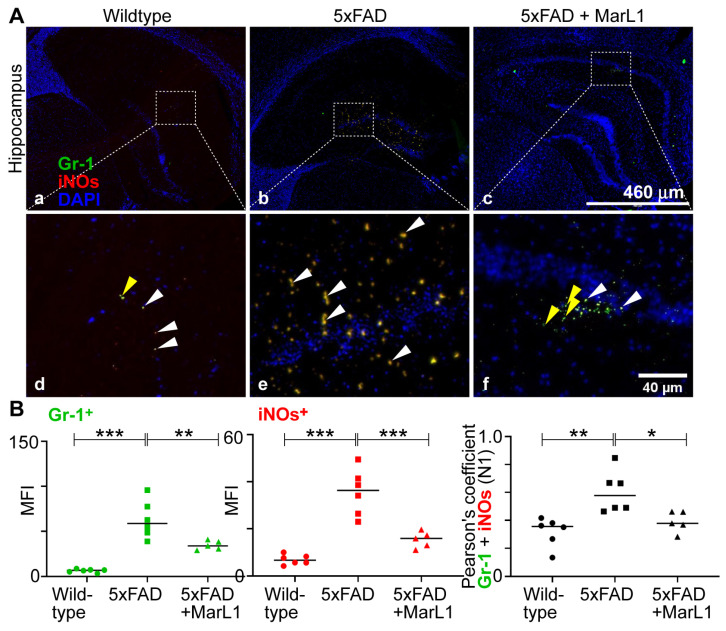
MarL1 treatment suppressed pro-inflammatory N1 polarization of neutrophils infiltrated into AD-pathogenic brains in 5xFAD mice. (**A**) Immunostaining of Gr-1 (green) for neutrophils and iNOs (red), an inflammatory marker. Panels a–c show hippocampus (4× magnification; scale bar: 460 µm). Panels d–f show zoomed-in images; scale bar: 40 µm. White arrows mark some Gr-1^+^iNOs^+^ cells and yellow arrows mark only Gr-1-positive cells in zoomed-in panels. (**B**) Quantification of Gr-1^+^ and iNOs^+^ in hippocampus. Left: MFI of Gr-1^+^; middle: MFI of iNOs^+^; right: Pearson’s coefficient for quantification of co-localization of Gr-1 and iNOs. Data are means ± SEM. Wildtype *n* = 6, 5xFAD *n* = 6, and 5xFAD+MarL1 *n* = 5. *** *p* < 0.001, ** *p* <0.01, and * *p* < 0.05.

**Figure 7:**
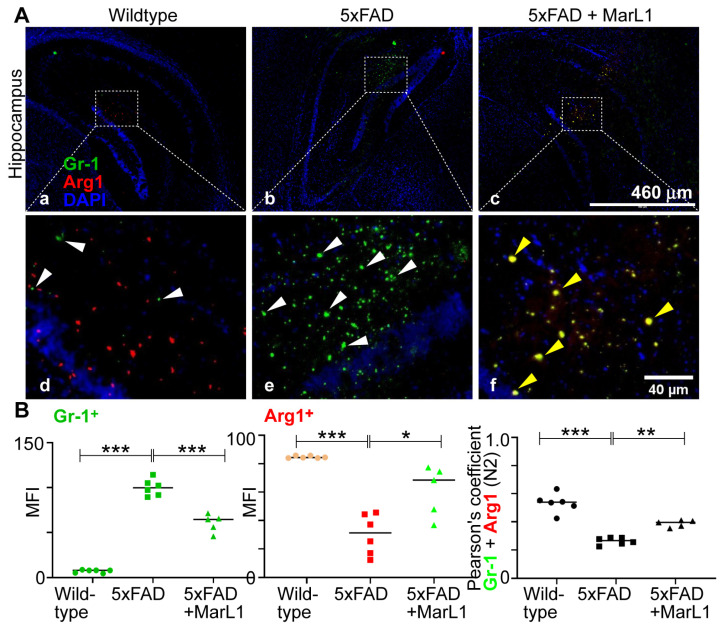
MarL1 treatment induced anti-inflammatory N2 phenotypic polarization of neutrophils infiltrated into AD-pathogenic brains in 5xFAD mice. (**A**) Immunostaining of Gr-1 (green) for neutrophils and Arg1 (red), an anti-inflammatory marker. Panels a–c show hippocampus (4× magnification, scale bar: 460 µm). Panels d–f show zoomed-in images; scale bar: 40 µm. White arrows mark Gr-1^+^ cells and yellow arrows mark Gr-1^+^Arg1^+^ cells in zoomed-in panels. (**B**) Quantification of Gr-1^+^ and Arg1^+^ in hippocampus. Left: MFI of Gr-1^+^; middle: MFI of Arg1^+^; right: Pearson’s coefficient for quantification of co-localization of Gr-1 and Arg1. Data are means ± SEM. Wildtype *n* = 6, 5xFAD *n* = 6, and 5xFAD+MarL1 *n* = 5. *** *p* < 0.001, ** *p* < 0.01, and * *p* < 0.05.

We also categorized the infiltrating neutrophil population into N1 neutrophils, a pro-inflammatory phenotype [39], and N2 neutrophils, an anti-inflammatory phenotype of pro-resolution of inflammation [40]. Immunostaining of coronal brain sections using the co-localization of Gr-1 and iNOS (Gr-1^+^iNOS^+^) revealed more Gr-1^+^iNOS^+^ N1 neutrophils in 5xFAD mice than in wildtype mice (Figure 6A), while MarL1 treatment reversed this trend in the hippocampal region. Pearson’s coefficients between the mean fluorescence intensities and MFIs of Gr-1^+^ cells and iNOS^+^ cells are consistent with this trend about the co-localization Gr-1^+^ and iNOS^+^ (Figure 6B right panel, *p* < 0.01 or 0.05). By contrast, the micrograph of Gr-1 and Arg1 co-localized brain sections showed fewer N2 neutrophils in 5xFAD mice than in wildtype mice (Figure 7A) and MarL1 treatment replenished the N2 population above that in vehicle-treated 5xFAD mice. This direct microscopic observation is consistent with the analysis of Pearson’s coefficients between MFIs of Gr-1^+^ cells and Arg1^+^ cells in the micrograph (Figure 7B right panel, *p* < 0.001 or 0.01). These histological results suggest that MarL1 treatment polarizes the shift of N1 to N2 neutrophil population in neurodegenerative events.

## 4. Discussion

Neuroinflammation is pivotal in the development of AD, as it can worsen Aβ and Tau pathologies [80,81,82]. In the present study, we examined the effect of MarL1 on the extent of inflammation in brains of transgenic 5xFAD mice.

We evaluated the histological effect of intranasal instillation of the MarL1 mediator from the age of 1.5 to 9 months in 5xFAD mice. Intranasal instillation of drugs is well recognized to partly bypass the BBB to deliver drugs to the brain more efficiently than *ip* or *iv* methods, thereby increasing drug bioavailability in the brain, while also delivering drugs noninvasively and to the blood circulation as well [43,83,84,85]. At the age of 12.5 months, 5xFAD mice show a significant reduction in levels of oligomeric Aβ42 and Aβ plaques in cortex and hippocampal regions of the brain and in the loss of NeuN^+^ neurons in CA3 and dentate gyrus regions of the hippocampus under MarL1 treatment (Figure 1). Quantification of the Aβ plaque numbers based on sizes greater than and less than 100 μm^2^ area [49,50,51] using thioflavin S staining revealed a marked reduction in the number of Aβ plaques following MarL1 treatment of 5xFAD mice (Figure S1). The neuroprotective effect of MarL1 was mediated by restoring the cholinergic neurons in striatum and decreasing the apoptotic cleaved caspase-3^+^ neurons in brain. Other studies have shown increased activation of cleaved caspase-3 in the hippocampus of AD patients and increases in the levels of synaptic pro-caspase-3 and cleaved caspase-3 in the postsynaptic density fractions [86]. These findings suggest that MarL1 has a neuroprotective effect in the brain. Aside from cholinergic neurons, GABAergic interneurons are also significantly involved in the etiology of Alzheimer’s disease and reduced in 5xFAD mice compared to wildtype mice [87]. The effects of MarL1 treatment on different interneurons in the striatum and other brain regions will be further investigated in future studies.

Neuroinflammation plays a vital role in neurodegeneration by contributing to neuronal damage and synaptic loss, with microglia as the key players [88,89,90]. Histological examinations of AD brains show that microglial cells are found in close association with Aβ deposits, especially dense-core plaques [91,92,93]. The quantity of microglia increases proportionally with plaque dimension [94]. Aβ deposition has been reported to attract a microglial cells, resulting in their accumulation at the periphery of the plaque [91]. Our results demonstrate enhanced microglial phagocytosis of Aβ plaques (Figure S2), and we found microglia in clusters in 5xFAD mice. These aggregates showed that microglial accumulation and proliferation in the brain could cause neuroinflammation, while MarL1 treatment reduced the Aβ accumulation, thereby decreasing the microglial activation under histological observation.

Enhanced microglial activation leads to increases in the expression of pro-inflammatory markers, including interleukin-1β, TNF-α, and iNOs, which in turn promote neuroinflammation [95,96]. Our data demonstrate that MarL1 treatment attenuated microglial activation by decreasing the population of Iba-1^+^ microglia and CD68 expression in the hippocampus (CA1, dentate gyrus) of the 5xFAD mouse brain. We phenotypically characterized microglia and calculated the number of microglia in different states as (1) ramified, (2) partially ramified, (3) partially amoeboid, (4) and fully amoeboid microglia [97,98]. We found that MarL1 reduced the level of amoeboid microglia, suggesting its inflammation-resolving and anti-inflammatory properties are a consequence of suppression of microglial activation (Figure 3).

Specialized pro-resolving lipid mediators are reported to modulate immunity and inflammation by resolving inflammation by triggering a biochemical paradigm shift commonly referred to as the “lipid mediator class switch” and skewing the M1/M2 macrophage balance toward the anti-inflammatory M2 phenotype, with replacement of injured cells and restoration of the normal functions of tissues [10]. Consistent with these reports, MarL1 treatment caused a shift in the M1/M2 population in brain as indicated by a surging Arg-1^+^ microglial population in the brain (Figure 4). These histological findings suggest that MarL1 is an effective immunoresolvent in brains.

In this study we explored the concept of neutrophil migration in brain parenchyma by immunohistological approaches. We observed the phenomenon of “neutrophil swarming” [99] in the cortex, as shown in Figure 5, representing neutrophil aggregation in the 5xFAD mouse brain. This is the first immunohistochemical report of neutrophil swarming in the brain cortex in the literature. Co-staining of Gr-1 (a marker for neutrophils) and claudin-5 (a marker for vasculature) showed that Gr-1^+^ neutrophils migrated into the brain and formed swarms, as neutrophils are nonresident cells in brains. We could not find neutrophil swarms in the brains of MarL1-treated 5xFAD mice, although neutrophils clusters that were not swarming were more evident than in wildtype mice. No swarms were detected in the brains of the wildtype littermates. These findings suggest that neutrophil infiltration into brains could contribute to AD pathogenesis since neutrophils produce reactive oxygen species and degradation enzymes that can cause neuroinflammation and neurodegeneration [38,76,100]. Furthermore, our findings revealed that MarL1 treatment inhibited the AD-linked neutrophil infiltration and swarm in the brains of 5xFAD mice.

Neutrophils play significant roles in the pathogenesis of AD and is categorized into pro-inflammatory N1 [39] and anti-inflammatory N2 subpopulations [40]. In our study, we found that Gr-1^+^iNOS^+^ N1 neutrophils increased in 5xFAD mice. We also found that MarL1 treatment dampens the elevation of N1 neutrophils in the brain (Figure 6). Furthermore, Gr-1^+^Arg1^+^ N2 neutrophils were amplified with MarL1 treatment and were lower in 5xFAD mice than in wildtype controls (Figure 7). These results demonstrate that chronic MarL1 treatment polarizes a shift from N1 to N2 in 5xFAD mice, suggesting resolution of inflammation in the brain.

Taken together, our immunohistological results provide the first evidence that MarL1 was effective in inhibiting Aβ pathology in brain. We will validate our findings with additional comprehensive research methods in the future. In addition, the 5xFAD mouse model is an aggressive early-onset transgenic AD model [20,101,102,103,104]; therefore, the protection effects of MarL1 need to be explored using less aggressive AD models, including late-onset AD models [104,105]. Furthermore, the different doses of MarL1 and their safety should be evaluated for future clinical studies. Further research is needed to fully understand the mechanisms involved in the neuroprotection imparted by MarL1 in AD. MarL1-specific receptor targeted research could be a promising strategy for developing new treatments for this debilitating disease. Given the structure similarity between MarL1 and maresin1, their receptors may share some commonalities. However, due to their structure difference and unique properties, there may also be distinct receptors. Screening and identifying potential receptors will be a future direction of our research. Investigating MarL1’s function on the neuroinflammatory response with an antagonist in vitro and in vivo will provide solid data for the mechanism involved in inflammation resolution related to Alzheimer’s disease.

## 5. Conclusions

**Figure 8:**
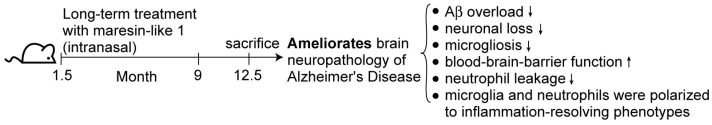
A graphic summary.

The long-term administration of MarL1 in 5xFAD mice had a positive impact on reducing the neuropathology associated with Alzheimer’s disease in the brains of 5xFAD mice—an animal model deemed appropriate for the study of early-onset AD and Aβ pathology. This study highlights the potential use of MarL1 as a therapeutic lead for the treatment of AD as shown in Figure 8. Further research is needed to fully understand the underlying mechanisms of action and to further optimize the treatment efficacy. MarL1 treatment efficiency and its involved mechanisms should also be studied using the animal models available for AD tau pathology.

## Data Availability

Data are contained within this article.

## Supplementary Materials

The following supporting information can be downloaded at: https://www.mdpi.com/article/10.3390/biomedicines12122865/s1, Figure S1: MarL1 treatment prevented or reduced the elevation of thioflavin S-positive cerebral plaques in brains of 5xFAD mice; Figure S2: The interaction between microglia (a–c) and amyloid beta plaques in cortex and hippocampus of 5xFAD mice.
