# Supplementary material for: Maresin-like 1 Ameliorates Neuropathology of Alzheimer’s Disease in Brains of a Transgenic Mouse Model
Source: Biomedicines. 2024 Dec 17;12(12):2865. doi: 10.3390/biomedicines12122865 (PMC11673747; doi:10.3390/biomedicines12122865)
Supplement: Supplementary file 1 [file biomedicines-12-02865-s001.zip › biomedicines-3245137-supplementary.pdf]

Supplemental Figures

Maresin-like 1 Ameliorates Neuropathology of Alzheimer's Disease in Brains of a Transgenic Mouse Model

Pallavi Shrivastava[1], Yan Lu[1], Shanchun Su[1], Yuichi Kobayashi[2, 3], Yuhai, Zhao[1], Nathan Lien[1], Abdul-Razak Masoud[1], Walter J. Lukiw[1,4], and Song Hong[1,4,*]

[1] Neuroscience Center of Excellence, School of Medicine, Louisiana State University Health New Orleans, 2020 Gravier St., New Orleans, LA 70112, USA

[2] Department of Bioengineering, Tokyo Institute of Technology, Box B-52, Nagatsuta-cho 4259, Midori-ku, Yokohama 226-8501, Japan

[3] Organization for the Strategic Coordination of Research and Intellectual Properties, Meiji University, 1-1-1 Higashimita, Tama-ku, Kawasaki 214-8571, Japan

[4] Department of Ophthalmology, School of Medicine, Louisiana State University Health New Orleans, 2020 Gravier St., New Orleans, LA 70112, USA

[*] Corresponding Authors: Song Hong (shong@lsuhsc.edu)

Keywords: Maresin-like, Alzheimer's Disease, neuroinflammation, neuropathogenesis, amyloid-β (Aβ), cholinergic neuron, cleaved-caspase-3, M1 or M2 microglia, N1 or N2 neutrophil

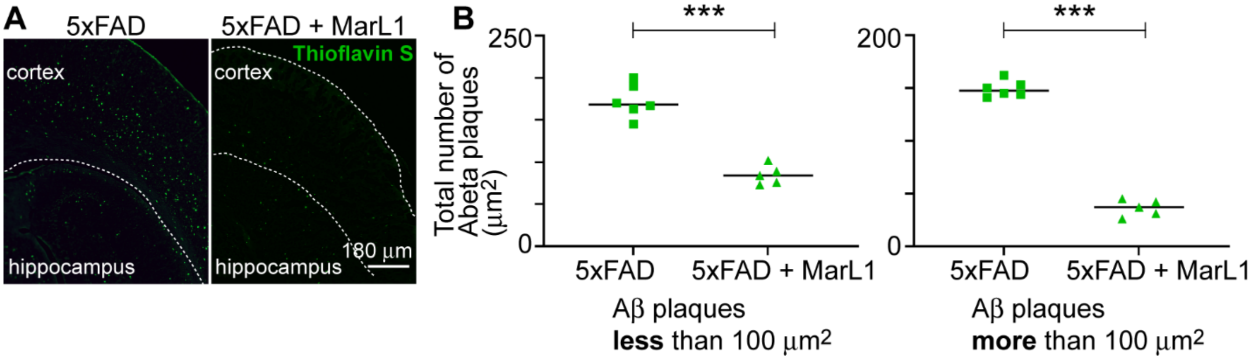

Figure S1. **MarL1 treatment prevented or reduced the elevation of thioflavin-S positive cerebral plaques in brains of 5xFAD mice**. (A) Thioflavin-S positive cerebral plaques in cortex and hippocampus. (B) The level of plaques was quantified in hippocampus and cortex in the brain, including number of plaques (size > 100 μm^2) and number of plaques (size < 100 μm^2). Data are Means ± SEM (n = 6 for 5xFAD, and n = 5 for 5xFAD + MarL1). ***$p < 0.001$.

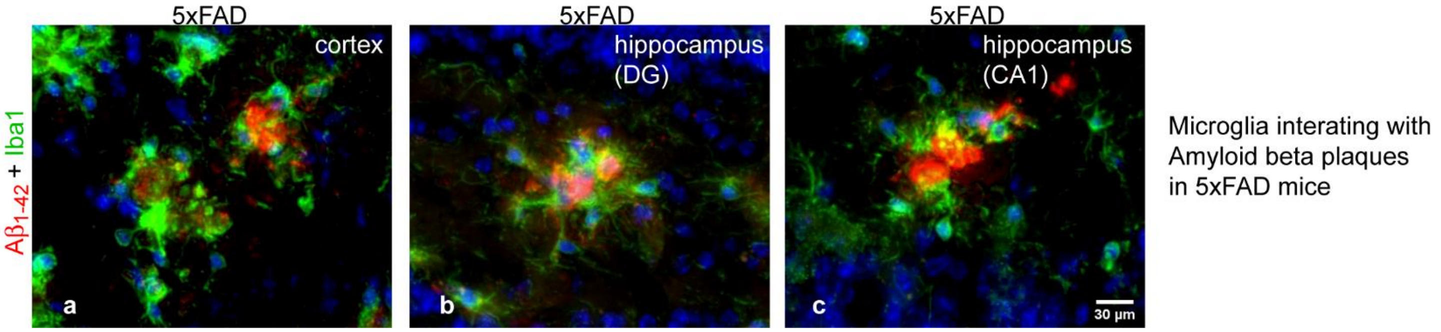

Figure S2. The interaction between microglia (a-c) and amyloid beta plaques in cortex and hippocampus of 5xFAD mice.
